# Hyperprogression of brain metastases following initiation of immune checkpoint inhibitors

**DOI:** 10.1007/s11060-025-04955-9

**Published:** 2025-02-07

**Authors:** Charissa A. C. Jessurun, Francesca Siddi, Noah L.A. Nawabi, Alexander F. C. Hulsbergen, Yu Tung Lo, Rohan Jha, Timothy R. Smith, Marike L. D. Broekman

**Affiliations:** 1 Computational Neuroscience Outcomes Center (CNOC), Department of Neurosurgery, Brigham and Women’s Hospital, Harvard Medical School, 75 Francis Street, Boston, Massachusetts 02115 USA; 2 Department of Neurosurgery, Leiden University Medical Center, Albinusdreef 2, 2333 ZA Leiden, Zuid-Holland The Netherlands; 3 Section of Neurosurgery, Department of Neurosciences, Biomedicine and Movement Sciences, University of Verona, Piazzale Stefani 1, 37124 Verona, Italy; 4 Department of Neurology, Massachusetts General Hospital, Harvard Medical School, 55 Fruit Street, Boston, Massachusetts 02114 USA; 5 Department of Neurosurgery, Haaglanden Medical Center, Lijnbaan 32, 2512 VA The Hague, The Netherlands

**Keywords:** Immune-checkpoint inhibitors, Hyperprogression, Brain metastases, Small cell lung cancer, Melanoma, Tumor growth rate

## Abstract

**Purpose:**

Immune checkpoint inhibitors (ICI) are increasingly being administered to cancer patients, including those with brain metastases (BMs). However, in a subset of cancer patients, ICI have shown to paradoxically accelerate tumor growth. This phenomenon is known as hyperprogressive disease (HPD). The aim of this study is to investigate the occurrence of HPD following initiation of ICI in BM patients.

**Methods:**

We retrospectively reviewed the charts of 60 surgically treated patients with BMs from non-small cell lung cancer or melanoma who were administered ICI at the Brigham and Women’s Hospital, Boston between July 2008 and July 2018. BM tumor volumes before and after initiation of ICI were collected. HPD was defined as a ‘post-immunotherapy’ tumor growth rate (TGR) > 2 times ‘pre-immunotherapy’ TGR within three months following initiation of ICI.

**Results:**

Among the 25 included patients treated with ICI, five patients showed HPD with an increase of post-immunotherapy TGR ranging from 4.9 to 207.7 times the pre-immunotherapy TGR. The median survival after initiation of ICI was was 8.0 months in the HPD cases and 13 months in the non-HPD patients.

**Conclusion:**

HPD occurred in about 20% of BM patients receiving ICI. More research is necessary to prospectively analyze the occurrence of HPD and identify predictive factors for HPD in BM patients.

**Supplementary Information:**

The online version contains supplementary material available at 10.1007/s11060-025-04955-9.

## Introduction

Immune checkpoint inhibitors (ICI) have proven to be a highly efficacious therapeutic option for cancer patients, including those with brain metastases (BM). These ICI target a multitude of immune checkpoints including programmed death 1 (PD-1), the ligand of PD-1 (PD-L1), or cytotoxic T-lymphocyte associated protein 4 (CTLA-4), resulting in prolonged activation of T-cell responses and subsequently antitumor activity [1]. Intracranial response rates range from 6 to 25% in melanoma BM patients treated with ICI monotherapy [2–6], 33% in non-small cell lung cancer (NSCLC) patients [7], and 53–57% in asymptomatic melanoma BM patients treated with a combination of anti-CTLA-4 and anti-PD-1 ICI [8, 9], although symptomatic BM patients show lower efficacy but responders could derive long-term benefit [8, 10]. The preliminary data from the anti-PD-1 brain collaboration (ABC) clinical trial showed a 5-year intracranial progression free survival (IC-PFS) of 46% and 5-year overall survival of 51% with combination therapy compared with only 15% and 34% with anti-PD-1 ICI, respectively [11]. ICI in combination with stereotactic radiosurgery shows even higher intracranial response rates and improved survival when compared to ICI alone [12–15]. 

However, a subset of patients have been reported to experience rapid increase in both tumor size and growth rate following initiation of ICI in comparison to pre-treatment kinetics, a phenomenon termed hyperprogressive disease (HPD) [16–24]. The reported extracranial incidence of HPD varies from 9 to 29% [16–24]. HPD has been associated with a poor prognosis and does not subsequently regress, in contrast to the radiographically diagnosed phenomenon of pseudoprogression [23, 25]. The median overall survival of patients who develop HPD is estimated to be about 3 months [16]. While most studies reporting on HPD have focused on the growth of existing tumors, the development of new lesions, including brain metastases, has been described in case reports as a possible sequelae of HPD as well [18, 20]. As intracranial HPD can rapidly result in mass effect and neurological deterioration, understanding its development in the brain after ICI initiation is of importance. In the present study, we report on the occurrence of intracranial HPD in surgically treated brain metastases patients receiving ICI.

## Methods

### Patient selection

Patients were retrospectively identified from the Brigham and Women’s Hospital institutional database of BM patients that underwent BM resection, and were eligible for inclusion in the present study based on the following characteristics: (1) at least one dose of PD-(L)1 inhibitors or CTLA-4 inhibitors with or without other concurrent chemotherapy or other immunotherapy between January 2008 and January 2018, (2) available magnetic resonance imaging (MRI) of the brain within 3 months before and after initiation of PD-(L)1 or CTLA-4 blockade. As ICI were initially most commonly administered to non-small cell lung carcinoma (NSCLC) and melanoma patients, we focused on BM patients with primary NSCLC and melanoma. Patients with incomplete records and/or those without available scans for brain tumor volume measurement were excluded.

### Data collection and analyzes

Demographical patient data (age at BM diagnosis and gender), tumor characteristics (tumor volume, location and primary tumor histology), and treatment characteristics (craniotomy, type of radiation therapy) were collected. The primary outcome of this study was the occurrence of HPD. The secondary outcome was overall survival (OS).

HPD was defined as time-to-treatment failure (TTF) of less than 3 months and post-immunotherapy tumor growth rate (TGR) more than twice that of pre-immunotherapy TGR [18, 19, 24]. TTF was defined as the interval between the initiation of ICI and radiological progression. TGR was calculated with the following formula: Δ tumor volume / Δ time (in months) [17, 18, 24]. Tumor volume was calculated by measuring the maximal left-right and anterior-posterior diameters (in cm) of the largest lesion on axial magnetic resonance imaging (MRI) slices, and the maximal cranio-caudal diameter (in cm) of the same lesion on coronal or sagittal slices, and applying the (AxBxC)/2 formula (this method has previously been used to calculate size of brain tumors and hemorrhages) [26, 27]. To differentiate HPD from pseudoprogression, the histological diagnosis after surgery was collected if available and the last follow-up MRI scans post-immunotherapy initiation were followed to observe if the tumor got smaller.

Descriptive statistics were used to report on the baseline variables. The median survival was calculated from the date of ICI initiation until death due to any cause or last date of follow-up.

## Results

### Patient selection

Between January 2008 to January 2018, 60 consecutive patients with brain metastases who underwent neurosurgical resection of BMs and received at least one dose of a PD-(L)1 inhibitor or CTLA-4 inhibitor at Brigham and Women’s Hospital were identified. Of these patients, 25 patients met inclusion criteria based on timing of first ICI dose and the availability of brain imaging. The median age of all patients was 62. The primary tumor was melanoma in 17 patients and NSCLC in 8 patients. Most patients received an anti-CTLA4 inhibitor (*n* = 12), 11 patients received an anti-PD(L) inhibitor and 2 patients received a combination of an anti-PD(L)1 and anti-CTLA-4 inhibitor. About 40% (*n* = 10) of the patients received previous stereotactic radiosurgery (SRS) to one or more lesions (Table 1).

**Table 1:** Baseline characteristics of 25 patients with BM that underwent neurosurgical resection and received at least one dose of a PD-(L)1 inhibitor or CTLA-4 inhibitor

	Total	HPD Cases
Gender	Female: 11 Male: 14	Female 3 Male 2
Median age at BM diagnosis	62	62
Primary tumor	Melanoma: 17 Lung: 8	Melanoma: 4 Lung: 1
Anti-PD(L)1	11	2
Anti-CTLA4	12	2
Anti-PD(L)1 + anti-CTLA-4	2	1
Previous SRS before ICI initiation	10	5

BM: brain metastasis; PD(L)1: programmed death (ligand) 1; CTLA4: Cytotoxic T-lymphocyte associated protein 4; HPD: hyperprogressive disease; SRS: stereotactic radiosurgery

**Table 2:** Patient, treatment and outcome characteristics of the hyperprogressive disease cases

Case	Age at BM diagnosis	Gender	Primary tumor	Type of ICI	Time from radiation to ICI (in months)	Time from ICI to surgery (in days)	Location lesion	Time to death (in months)	TTF (in days)	TGR pre-ICI	TGR post-ICI
1	65	Female	Melanoma	Pembrolizumab	21 (SRS)	22	Right temporal lobe	1	5	0.02	0.40
2	66	Male	NSCLC	Nivolumab	14 (SRS)	30	Left cerebellum	5	8	0.01	0.18
3	29	Male	Melanoma	Ipilimumab and nivolumab	17 (WBRT)	8	Right inferior temporal lobe	33	6	0.14	0.68
4	58	Female	Melanoma	Ipilimumab	3 (SRS)	-	Right frontal lobe	24	82	0.004	0.11
5	62	Female	Melanoma	Ipilimumab	-	76	Right parietal lobe	8	74	0.06	11.45

BM: Brain metastases; ICI: Immune checkpoint inhibitors; TTF: time to treatment failure; TGR: Tumor growth rate; SRS: Stereotactic radiosurgery; WBRT: Whole brain radiation therapy; NSCLC: Non-small cell lung cancer. Time to death was calculated from the date of ICI initiation until death due to any cause

### Hyperprogressive disease cases

**Fig. 1:**
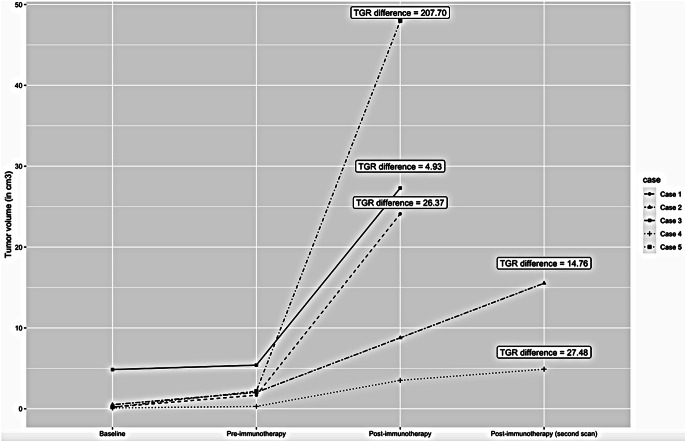
Line chart showing a comparison of tumor volumes (cm^3^) and the difference in tumor growth rate (TGR) of the five hyperprogressive disease cases before and after initiation of immune checkpoint inhibitors (ICI). Case 1 showed a TGR difference of 26.37, case 2 showed a TGR difference of 14.76, case 3 showed a TGR difference of 4.93, case 4 showed a TGR difference of 27.48, and case 5 showed a TGR difference of 207.70

Of the 25 included patients, five (20%) showed HPD of existing lesions. Three patients had more than one BM, however none experienced HPD of more than one lesion. The cases showed an increase of post-immunotherapy TGR ranging from 4.9 to 207.7 times the pre-immunotherapy TGR (Fig. 1). The primary tumor was melanoma in four of the HPD cases and NSCLC in one. The median age of the cases was 62 years at diagnosis, and two patients were male. Two patients received a CTLA-4 inhibitor, two patients received a PD-1 inhibitor, and one patient received combination therapy with anti-PD1 and anti-CTLA-4 (Table 2). Detailed case descriptions and MRI images of the hyperprogressive disease cases can be found in the supplementary material 1 and 2.

All cases received radiation therapy for BM before initiation of ICI therapy, however only three patients received SRS to the hyperprogressive lesion 3–21 months prior to ICI initiation, one patient received WBRT 17 months prior to ICI initiation, and one patient did not receive any radiation to the hyperprogressive lesion.

Four of the five cases underwent resection of the hyperprogressive lesion 8–76 days after ICI initiation. All pathology reports described metastatic disease, although one report also described extensive necrosis but conclusively true progression (case 2), and one report described extensive reactive changes and treatment effect in the surrounding brain tissue next to metastatic disease (case 3).

### Survival

The median survival after initiation of ICI of the HPD patients was 8.0 months; the median survival of the patients who did not develop HPD was 13.0 months. Due to the low number of HPD cases, no further statistical analyzes were performed.

## Discussion

Of the 25 BM patients treated with surgery and ICI, five patients experienced HPD (20%). The HPD patients showed an increase of post-immunotherapy TGR ranging from 4.9 to 207.7 times the pre-immunotherapy TGR. The patients that developed HPD had a shorter median OS in comparison to those that did not, although no comparative analysis was possible due to the low number of cases (8.0 versus 13.0 months, respectively).

HPD after initiation of ICI has been described in several types of cancers, including melanoma, lung, renal, colorectal, urothelial and gastric cancer [28]. However, much controversy exists on this relatively new phenomenon, as it can occur in a wide variety of cancer with vastly different biologic and molecular characteristics. It is difficult to attribute HPD to ICI therapy, as the rapid increase in TGR may be secondary to factors other than the checkpoint blockade [17, 29]. There is a possibility that the rapid growth observed in these patients could be a part of the natural course of their disease, and that the administration of immunotherapy may be merely coincidental. However, the current definition of HPD attempts to account for the natural course of disease by using tumor size and growth rate prior to ICI initiation as a control. According to a recent review, several ways to measure the tumor growth are described in literature: (1) TGR ratio comparing the speed of increase in tumor volume before and after ICI therapy (utilized in the present study); (2) tumor growth kinetics (TGK) ratio comparing the speed of increase in tumor size before and after ICI therapy; (3) early tumor burden increase between baseline imaging and the first time point after treatment; and (4) a combination of the aforementioned methods [30]. Some studies also consider the development of new lesions as HPD.

Moreover, HPD has been observed following the initiation of other treatment modalities including chemotherapy [17], RAF inhibition in KRAS mutated NSCLC, and crizotinib in anaplastic lymphoma [31, 32]. Treatment of BM with chemotherapy is inherently challenging due to the blood-brain barrier preventing anticancer drug delivery [33], and thus the possibility of experiencing HPD following administration of these treatments in brain tumors may be minimized compared to other cancers. However, ICI therapy has shown to exert intracranial efficacy in brain metastases [2–5, 7], highlighting the importance of mapping the occurrence of HPD in BM.

The mechanism of HPD is still largely unknown, however several mechanisms have been postulated. A recent study by Zang et al. [34] classified the possible mechanisms into three categories: intrinsic immunological reasons, acquired resistance, and possible external factors. PD-1-positive regulatory T cells (Tregs), and alternatively activated macrophages, seem to play an important role in HPD caused by PD-1 inhibitors as higher infiltration of both are seen in HPD patients in comparison with non-HPD patients [35–37]. Moreover, cancer stem cells, cells in tumors that possess characteristics including self-renewal and continuous proliferation and the ability to differentiate [38], may contribute to the acquired resistance hypothesis as cytotoxic T lymphocytes might induce cancer cell stemness [39]. Understanding the mechanism of HPD is critical, as doing so will allow clinicians to identify biomarkers that may predict its occurrence. To date, several predictive biomarkers for the development of HPD have been evaluated, such as MDM2 amplification, EGFR mutations, cell free DNA chromosomal number instability, baseline highly differentiated CD28- CD27- CD4 T cells, and neutrophil to lymphocyte ratio. However, none of these are currently used in clinical practice [40]. Although tumor PD-L1 expression is a potential predictive biomarker for response to anti-PD-(L)1 therapy, it is not associated with HPD [40]. 

Contrasting evidence exists regarding the effects of previous therapy on the risk for HPD development. Studies like that by Champiat et al. have failed to demonstrate an association between previous therapies, including chemotherapy, and the development of HPD [16]. However, other studies have suggested that previous irradiation may enhance the risk of HPD [19], possibly owing to radiation-induced changes in tumor microenvironment. In the present series, four out of five patients (80%) who developed HPD had received previous brain irradiation.

It is difficult to distinguish HPD from pseudoprogression and/or treatment effects including radiation necrosis, as the current criteria for HPD are based on radiological measurements. Pseudoprogression is defined as lack of true tumor progression in the setting of an increase in tumor size and subsequent tumor regression [41]. Radiation necrosis is a delayed complication of radiation therapy for brain tumors and head and neck cancers with necrotic degradation of brain tissue with a reported incidence of 0 to 30% [42]. The gold standard for diagnosis of both pseudoprogression and radiation necrosis is histological confirmation, however imaging techniques can help distinguish pseudoprogression and radiation necrosis from tumor progression. In four patients that underwent resection of the proposed hyperprogressive lesion, the intra-operatively collected tissue samples demonstrated metastatic disease tumor cells. However, one report also described extensive necrosis but conclusively true progression, and one report described extensive reactive changes and treatment effect in the surrounding brain tissue next to metastatic disease. This is in contrast to the defined characteristics of histopathological biopsies of pseudoprogression lesions, which show infiltration and recruitment of various immune cells in the tumor [43, 44], however radiation necrosis might have been at least partly responsible for the rapid growth seen in these two cases that received prior SRS. In addition, the clinical course of the cases favored hyperprogression, as none of them experienced tumor regression, and all experienced rapid tumor growth shortly after ICI administration. Of note, one patient died nine days after surgery of the hyperprogressive brain lesion. Pseudoprogression is often accompanied with an improved general condition upon regression of the tumor, whereas a deteriorating general condition may indicate true progression or hyperprogression [41]. 

This study has several limitations to consider. Firstly, this study was retrospective in nature, and examined only a small and highly selective number of patients who underwent neurosurgical resection of BM and received ICI. The database used to identify these patients, unfortunately, did not include non-surgically treated patients with BM which has introduced significant selection bias to the study population. Our sample size was further limited by the fact that several patients with likely HPD lesions did not undergo resection and subsequent pathological analysis to confirm their diagnosis of expansile BM.

The present study is the first to specifically assess the occurrence of HPD in brain metastases in patients receiving ICI, to our knowledge. There is a need for further research into the occurrence of HPD in BM patients receiving ICI. Studies should aim to prospectively compare tumor growth in BM patients after starting ICI, to identify predictive factors for HPD in BM patients, and to analyze the pathology pattern in radiologically diagnosed HPD patients. In addition, it would be interesting to compare the occurrence of HPD in ICI-treated patients with non-ICI treated patients, surgically and non-surgically treated patients, or patients receiving different therapies including radiotherapy, targeted therapy or chemotherapy. Doing so will facilitate the ability to offer personalized treatment to patients experiencing HPD.

## Conclusions

Possible hyperprogression of brain lesion(s) occurred in 20% of the NSCLC and melanoma BM patients receiving ICI in this study. More research is necessary to prospectively analyze the occurrence of HPD in BM patients receiving ICI in combination with different treatment modalities, and identify predictive factors for HPD.

## Electronic supplementary material

Below is the link to the electronic supplementary material.

Supplementary Material 1

## Data Availability

The datasets generated during and/or analysed during the current study are available from the corresponding author on reasonable request.
