# Supplementary material for: Hyperprogression of brain metastases following initiation of immune checkpoint inhibitors
Source: J Neurooncol. 2025 Feb 7;172(3):667–73. doi: 10.1007/s11060-025-04955-9 (PMC11968457; doi:10.1007/s11060-025-04955-9)
Supplement: Supplementary file 1 — Supplementary Material 1 [file 11060_2025_4955_MOESM1_ESM.docx]

**Supplementary material 1**

**Case descriptions leading up to possible hyperprogression of BMs**

**Case 1**

The patient presented with acute confusion three weeks after initiation of pembrolizumab, and the MRI brain revealed a significantly enlarged lesion in the right temporal lobe and a haemorrhagic right frontal (TGR difference of 26.37). The patient underwent resection of the right temporal hyperprogressive lesion 22 days after initiation of pembrolizumab, however, nine days post-surgery the patient passed away due to the development of haemorrhage in multiple new lesions distant to the resection cavity. The pathology report described malignant melanoma.

**Case 2**

Routine imaging showed enlargement of the left cerebellar tumor (TGR difference of 14.76) with significant mass effect without any symptoms after the first dose of nivolumab. The patient underwent left suboccipital craniotomy which showed tumor tissue and extensive necrosis but conclusively metastatic disease/true progression. About three months later further systemic and intracranial disease progression was noted in the form of two new metastases in the deep right frontal lobe, as well as local recurrence in the left cerebellum resection cavity, after which the patient passed away.

**Case 3**

One week following initiation of ipilimumab and nivolumab the patient developed left hemiparesis, and volume measurement of the right temporal lesion revealed a 4.9 fold increase in the TGR post-immunotherapy compared with TGR pre-immunotherapy. The patient underwent surgical resection of the right temporal BM which showed metastatic melanoma and extensive reactive changes and treatment effect in the surrounding brain tissue. Shortly after surgical resection ipilimumab and nivolumab were discontinued due to hepatitis. Multiple recurrences of intracranial disease were noted and patient passed away 33 months after initiation of ICI therapy.

**Case 4**

Three months after initiation of ipilimumab the MRI showed progression of the right frontotemporal lesion with associated edema in addition to new metastatic disease although the patient was clinically stable at that time. The patient underwent SRS two weeks later. The patient had multiple recurrences of intracranial disease and passed away 24 months after initiation of ICI therapy.

**Case 5**

The patient was admitted about two months after the start of ipilimumab with significant left-sided upper and lower-extremity weakness with a massive increase in size of the right parietal lesion on MRI. The patient underwent resection of the lesion which showed metastatic melanoma. The patient continued to decline with new intracranial lesions and passed away 8 months after ICI initiation.

**Supplementary material 2**

| **HPD Cases** | **Baseline** | **Pre-immunotherapy** | **Post-immunotherapy** | **Post-immunotherapy (second scan)** | **Last follow-up scan** |
| --- | --- | --- | --- | --- | --- |
| **1** |  |  |  |  |  |
|  | -58 days | -9 days | +5 days | +20 days (postoperative) | +21 days |
| **2** |  |  |  |  |  |
|  | -112 days | -49 days | +8 days | +27 days | +123 days (postoperative) |
| **3** |  |  |  |  |  |
|  | -12 days | -10 days | +6 days | + 9 days (postoperative) | + 970 days |
| 4 |  |  |  |  |  |
|  | -80 days | -30 days | +82 days | +95 days | +588 days (after SRS) |
| 5 |  |  |  |  |  |
|  | -50 days | -14 days | +74 days | + 74 days (postoperative) | +253 days |

**MRI images of the presumed hyperprogressive disease (HPD) lesions.** 1) a right temporal brain lesion of case 1 a number of days before and after initiation of pembrolizumab of which the second post-immunotherapy scan is after stereotactic radiosurgery (SRS) and the last follow-up scan available, 2) a left cerebellar lesion of case 2 a number of days before and after initiation of nivolumab and the last follow-up scan available and postoperative, 3) a right inferior temporal lobe brain lesion of case 3 a number of days before and after initiation of nivolumab and ipilimumab of which the second post-immunotherapy scan is postoperative and the last follow-up scan available, 4) a right frontal lobe brain lesion of case 4 a number of days before and after initiation of ipilimumab and the last follow-up scan available that is after several SRS sessions, 5) a right parietal brain lesion of case 5 a number of days before and after initiation of ipilimumab of which the second post-immunotherapy scan is postoperative, and the last follow-up scan available
